# Aortic Wave Dynamics and Its Influence on Left Ventricular Workload

**DOI:** 10.1371/journal.pone.0023106

**Published:** 2011-08-11

**Authors:** Niema M. Pahlevan, Morteza Gharib

**Affiliations:** 1 Option of Bioengineering, Division of Engineering & Applied Sciences, California Institute of Technology, Pasadena, California, United States of America; 2 Graduate Aerospace Laboratories, Division of Engineering & Applied Sciences, California Institute of Technology, Pasadena, California, United States of America; Harvard University, United States of America

## Abstract

The pumping mechanism of the heart is pulsatile, so the heart generates pulsatile flow that enters into the compliant aorta in the form of pressure and flow waves. We hypothesized that there exists a specific heart rate at which the external left ventricular (LV) power is minimized. To test this hypothesis, we used a computational model to explore the effects of heart rate (HR) and aortic rigidity on left ventricular (LV) power requirement. While both mean and pulsatile parts of the pressure play an important role in LV power requirement elevation, at higher rigidities the effect of pulsatility becomes more dominant. For any given aortic rigidity, there exists an optimum HR that minimizes the LV power requirement at a given cardiac output. The optimum HR shifts to higher values as the aorta becomes more rigid. To conclude, there is an optimum condition for aortic waves that minimizes the LV pulsatile load and consequently the total LV workload.

## Introduction

Congestive heart failure (CHF) has reached an epidemic proportion in the US and worldwide with serious consequences in terms of human suffering and economic impact. In the US alone, there are 60,000 patients dying each year with CHF as the underlying cause. Approximately 5,800,000 Americans have been diagnosed with this condition and this number is increasing every year [1]. In the absence of myocardial infarction, hypertension is a primary risk factor of CHF [2] mainly due to the chronic elevation of the left ventricular (LV) workload and the development of left ventricular hypertrophy (LVH) [3], [4], [5].

Pulsatile flow generated by the heart enters the compliant aorta as pressure and flow waves. These waves propagate and reflect throughout arterial vasculature, thus playing a dominant role in the hemodynamics of the arterial system. The hemodynamic load on the heart has two parts: steady and pulsatile. The steady load is the result of the resistance from the arterial network to the mean part of the flow. The pulsatile load depends on the interaction between the heart's pumping characteristics (stroke volume, heart rate, and ejection fraction) and arterial wave dynamics. Significant efforts have been made in the past to elucidate the role of wave reflections in heart failure [6], [7], [8]. Clinical studies have confirmed that abnormal pulsatile loads play an important role in the pathogenesis of left ventricular hypertrophy (LVH) and CHF [4], [9], [10]. O'Rourke [11] suggested four important factors that control the level of the pulsatile load applied to the heart: 1-rigidity of the aorta and other large vessels, 2-interaction between the left ventricular and the terminal of the vasculature in the upper and lower parts of the body, 3-wave reflection, and 4-balance between the heart rate and the body length. He showed in an animal (dog) study that the ratio of the pulsatile load over the total load decreased as the heart rate increased, and that the ratio increased as the aortic distensibility decreased. However, previous studies did not investigate the interplay between aortic rigidity and heart rate (HR) [11].

The wave dynamics in a compliant tube is controlled by the fundamental frequency of the propagating waves, the material properties of the tube, and wave reflections [12], [13]. Similarly, aortic wave dynamics depend on heart rate, aortic compliance, and the locations of reflection sites. We hypothesized that there exists a specific heart rate at which LV pulsatile load becomes a minimum for a given physiological condition.

To test our hypothesis, we implemented a computational approach in order to be able to examine a large spectrum of wave states. This approach enabled us to study aortic rigidity and HR while having a better control on parameters such as the aortic input flow wave (ventricular ejection wave), terminal compliance, peripheral resistance (PR), cardiac output (CO), and the locations of reflection sites.

The goal of this study is to investigate the effects of different states of aortic wave dynamics on the LV power requirement (LVPR). Various states of aortic wave dynamics are produced by changing the heart rate and aortic wall rigidity (aortic compliance) while fixing other determinant factors of wave dynamics and power requirements.

## Methods

### Physical Model

**Figure 1:**
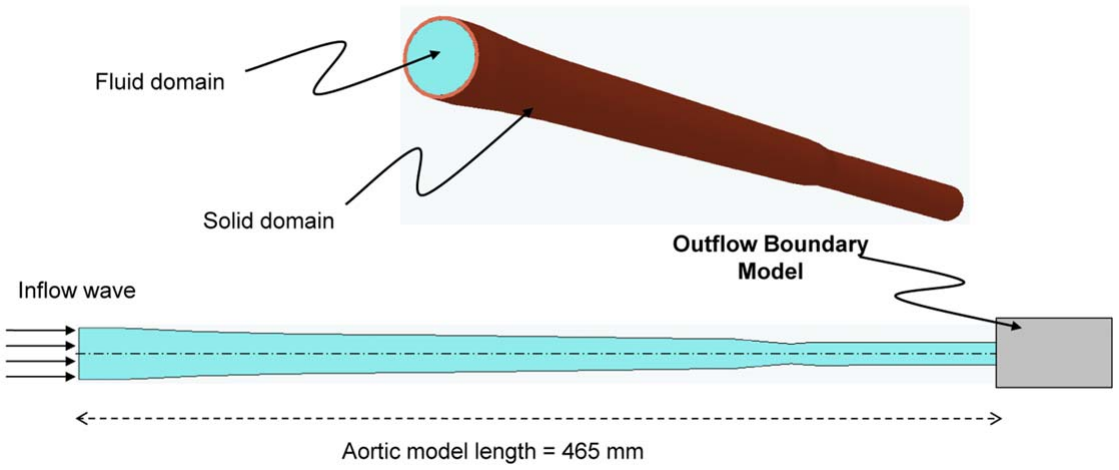
Computational model of the aorta.

A three-dimensional axisymmetric model of the aorta was used (Figure 1). The geometrical data of the aortic model, such as aortic length, inner diameter, and wall thickness, were within the average physiological range [14]. The aortic wall was assumed to be elastic and isotropic. These wall material assumptions are applicable when modeling large central arteries, especially the aorta, but it may not be suitable for radial arteries or arterioles since these vessels are more anisotropic and viscoelastic [15]. A further concern about our wall assumption might be the nonlinear dependency of the vessel's wall elasticity on the mean arterial pressure; however, for the normal mean pressure (less than 125 mmHg) the relation is linear [15]. The material properties of the wall were taken from Nichols *et al.* [15] The tapering and the change of wall stiffness along the aorta were considered, though the aortic arc and bifurcation were excluded since the model is 3D axisymmetric. The blood was assumed to be an incompressible Newtonian fluid, and the different levels of aortic rigidity considered were multiplicative factors of a minimum rigidity level *E_1_*(*x*) (*x* is the distance from the heart) of a healthy 30-year-old man taken from Nichols *et al.* [15]. The physical parameters of the aortic model are summarized in Table 1.

**Table 1:** Physical parameters*.

Physical parameters	Symbol	Value
Blood density	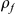	1050 *kg/m^3^*
Blood viscosity	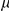	0.0051 *kg/m.sec*
Aortic wall density	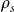	1050 *kg/m^3^*
Young's modulus of the sinus of Valsalva	*E_sin_*	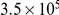 *Pa*
Young's modulus of the ascending aorta	*E_asc_*	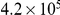 *Pa*
Young's modulus of the descending aorta	*E_des_*	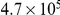 *Pa*
Young's modulus of the upper part of the abdominal aorta	*E_upab_*	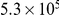 *Pa*
Young's modulus of the lower part of the abdominal aorta	*E_lowab_*	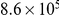 *Pa*
Aortic wall Poisson's ratio	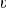	0.45
Wall thickness of the sinus of Valsalva	*h_sin_*	1.6 *mm*
Wall thickness of the ascending aorta	*h_asc_*	1.4 *mm*
Wall thickness of the descending aorta	*h_des_*	1 *mm*
Wall thickness of the upper part of the abdominal aorta	*h_upab_*	0.9 *mm*
Wall thickness of the lower part of the abdominal aorta	*h_lowab_*	0.8 *mm*

*Since the physical parameters vary in each aortic section, the average values within the sections have been given. The Young's modulus values in this Table are for the case of minimum rigidity (*E_1_*).

**Figure 2:**
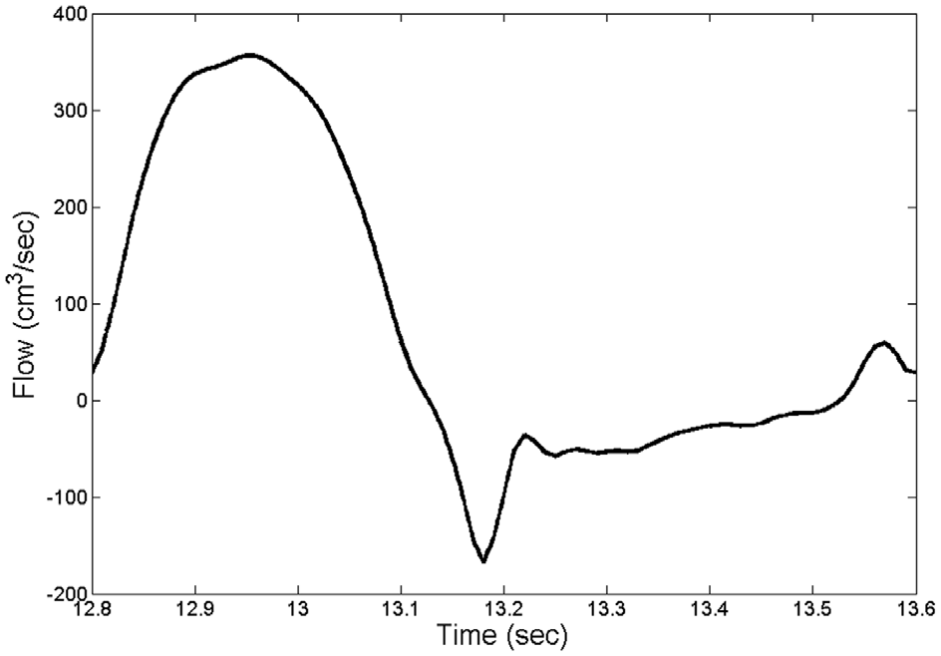
Inflow wave.

At the inlet, we imposed a physiological flow wave (Figure 2), from Matthys [16], with a flat velocity profile and scaled to give a cardiac output (CO) of 4.6 L/min for any given heart rate.

### Mathematical method

#### Solid Model

Large deformation with small strain theory was assumed for the formulation of elastic wall motion [17]. We assume the walls are composed of elastic isotropic material. Dynamic motion of the aortic wall was formulated by a constitutive relation for linear elastic isotropic materials and balance of momentum equations in Lagrangian form as [17], [18]

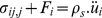
(1)

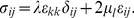
(2)Here, 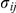 is the wall stress tensor, *F* is the external force, *u* is the displacement vector, 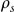 is the wall density, and *λ*, *μ_l_* are Lamé constants.

#### Fluid Model

To solve for pressure and flow fields in the fluid domain, we use the full Navier-Stokes (N-S) equations. In Eulerian form, they are
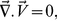
(3)

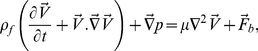
(4)where 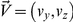 represents the flow velocity vector, 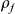 is the fluid density, *p* is the static pressure, *μ* is the dynamic viscosity, and 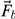 is the body force.

Since our fluid domain has moving boundaries, an arbitrary Lagrangian-Eulerian (ALE) formulation is used for the analysis of the fluid flow [19], [20], [21], [22]. This formulation can be directly coupled with the Lagrangian formulation of the solid domain. In an ALE formulation, the total time derivative (*d/dt*) for all the solution variables is given by [23]

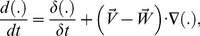
(5)where *δ(.)/δt* is the transient term at the mesh position, 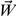 is the mesh velocity, and 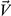 is the actual fluid particle velocity. Applying *equation 5* in *equation 4* gives [23]

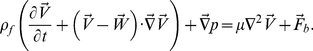
(6)


#### Coupling conditions

With no-slip boundary conditions at the wall, the coupling equations at the solid-fluid interface are
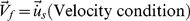
(7)

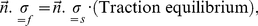
(8)where 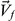 and 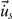 are the respective velocities of the fluid and solid at the interface, 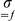 is fluid stress tensor, 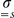 is solid stress tensor, and 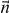 is the unit vector in the normal direction.

#### Boundary Conditions

A novel extension tube boundary model was used for the outflow boundary condition at the terminal of the abdominal aorta. This boundary model extends the computational domain with a straight elastic tube connected to a contracted rigid tube [24]. Parameters such as the ratio of the radii of the rigid tube together with the length and elasticity of the elastic boundary tube are selected to represent the effects of a truncated vascular network (resistance, compliance, and wave reflection) [24].

The parameters of the outflow boundary condition model are given in Table 2, where the contraction ratio is the ratio of the radius of the rigid boundary tube beyond the contraction to the original radius before the contraction. The parameters of outflow boundary condition were kept the same for all simulations.

**Table 2:** Outflow boundary parameters.

Description	value
The inner radius of the elastic boundary tube	6.31 *mm*
Total length of the elastic boundary tube	105 *mm*
Wall thickness of the elastic boundary tube	0.65 *mm*
Contraction ratio of the rigid boundary tube	0.76
Total volume compliance of the boundary model	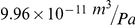

### Numerical method

A finite-element scheme was applied to solve the equations of the solid and fluid models incrementally in time using the commercial package ADINA 8.6 (ADINA R&D, Inc., MA). A direct two-way coupling fluid-structure interaction (FSI) method (simultaneous solution method) was used to couple the fluid and solid domains at the interface. In this method, the discretized fluid, solid, and coupling equations are all combined in one matrix [19]. In summary, the general computational steps in the employed direct coupling method were (i) to assemble the solid and fluid equations separately into a single fluid and single solid model; (ii) to assemble the solid matrix, fluid matrix, and the coupling matrices into one coupled matrix system; (iii) to solve the linearized equation of the coupled system and to update the solution; and (iv) to compute and check the residuals against the specified tolerance. If the solution did not converge, the process was restarted from step (i) [19], [24].

**Figure 3:**
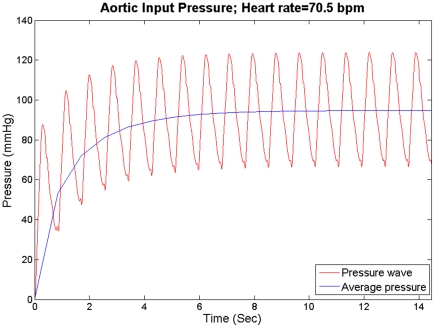
The mean pressure at the aortic input reaches a steady state for HR = 70.5 bpm.

An implicit Euler backward method with Newton-Raphson iteration was used for the time integration with a time step of 0.00125 s. A total of 2420 nine-node axisymmetric elements were used to mesh the solid domain, and a total of 17,416 three-node axisymmetric elements were used for the fluid domain. Further simulations with different time steps and mesh sizes confirmed that these results were independent of spatial and temporal discretizations. All simulations started from rest until the mean of the aortic input pressure reached a steady state as shown in Figure 3.

### Power Calculation

The total power (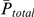) was calculated as the average of the product of the pressure (*p(t)*) and flow (*q(t)*) over a cardiac cycle. The steady power (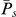) was the product of mean pressure (*p_mean_*) and mean flow (*q_mean_*), and the pulsatile power (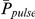) is the difference of the total power and steady power.
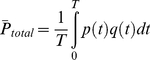
(12)

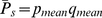
(13)

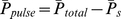
(14)


## Results

The simulations were run for seven different levels of aortic rigidities, ranging from a 30-year old healthy individual (*E_1_(x)*) to a 70-year old sick individual suffering from aortic stiffening (*E_7_(x)*) (values taken from Nichols *et al*) [15]. Here, *E_i_*(*x*) indicates that Young's modulus changes along the aorta, where *x* is the axial distance from aortic input. The different levels used were multiplicative factors of *E_1_*(*x*) (Table 1) as *E_2_ = 1.25E_1_*, *E_3_ = 1.5E_1_*, *E_4_ = 1.75 E_1_*, *E_5_ = 2E_1_*, *E_6_ = 2.5E_1_*, and *E_7_ = 3E_1_*. Each case of the aortic rigidity was run for eight heart rates (70.5, 75, 89.5, 100, 120, 136.4, 150, and 187.5 beats per minute (bpm)). In all simulations, CO, PR, the terminal compliance, and the shape of the inflow wave were kept constant.

### Pressure Wave Solution

**Figure 4:**
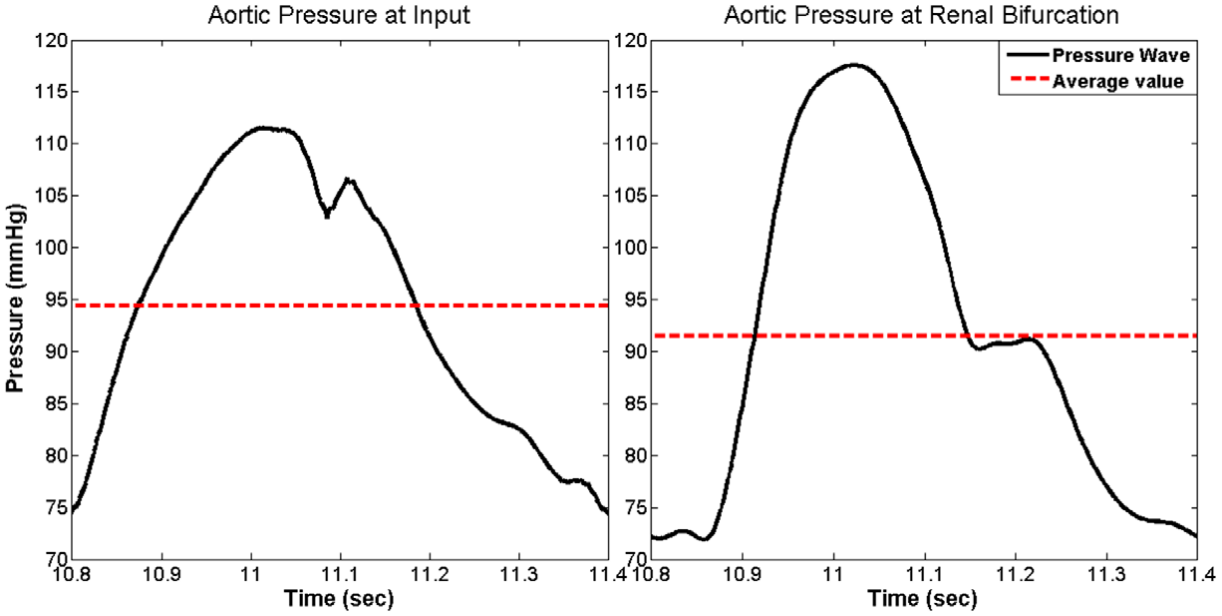
Simulated results from our aortic model showing pulse pressure amplification, pressure narrowing, shifting of the second-highest peak, and the existence of the dicrotic notch. The average per cycle decreases due to viscous losses, consistent with realistic results. Data is for a 100 bpm heart rate and aortic rigidity of *E_1_* taken during one cycle after reaching steady state.

To verify the model, we compared results with well-known features of aortic pressure waves [15], [25]: (i) the pulse pressure amplification; (ii) the narrowing of the pressure waves as they travel down the aorta; (iii) the existence of a dicrotic notch; and (iv) the shifting of the second-highest pressure peak to the end of the cycle. Figure 4 shows that our computational model captured all four characteristics of the pressure wave. The model additionally captured another important physical feature of the blood flow—the mean pressure decreased down the aorta (see Figure 4).

### Effects of Aortic Compliance on Left Ventricular Power Requirement

**Figure 5:**
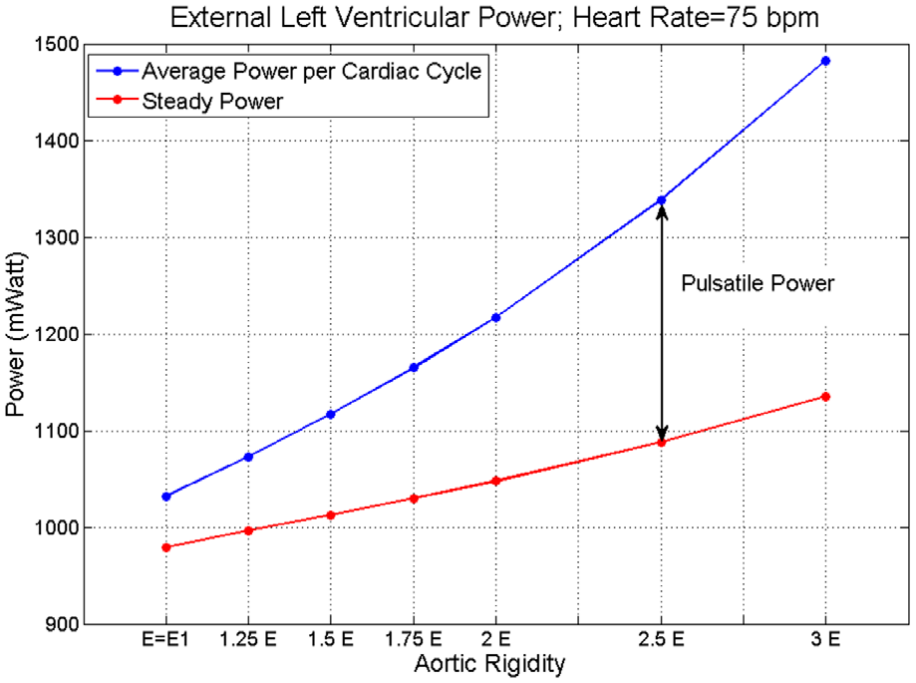
The blue line is average per cycle of the total external left ventricular power (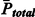) and the red line is the external steady power (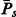) at different levels of aortic rigidities. The value of *E = E_1_* corresponds to the level of aortic rigidity given in Table 1. The difference between the two curves shown by the double arrow is pulsatile power (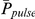). The results are for HR = 75 bpm.

Figure 5 shows average values per cycle for the total LV power requirement—external LV power (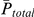) —and the steady power (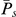) versus aortic rigidity for an HR of *75 bpm*. It shows that both 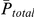 and 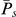, as well as the pulsatile power (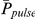 = 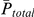
−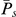), increased at higher rigidities. This is in agreement with clinical findings [26].

### Effect of Heart Rate on Left Ventricular Power Requirement

**Figure 6:**
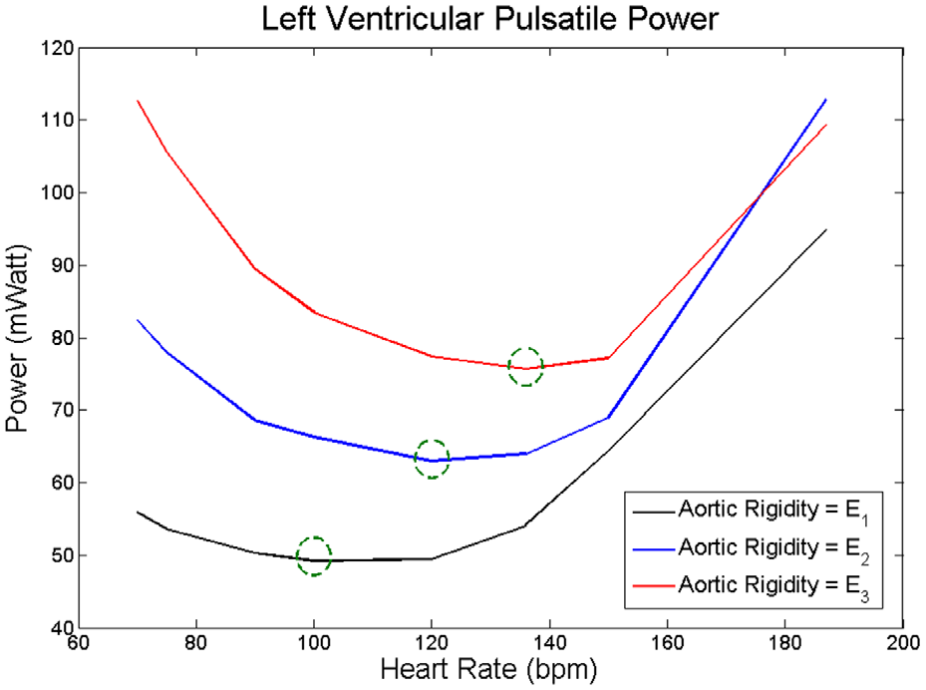
Average value per cycle of pulsatile power 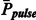 versus HR for three different levels of aortic rigidities. *E_1_* (black) corresponds to the values given in Table 1, *E_2_ = 1.25E_1_* (blue), and *E_3_ = 1.5E_1_* (red). Green circles are the heart rates corresponding to the minimum 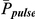 value at each level of aortic rigidity.

**Figure 7:**
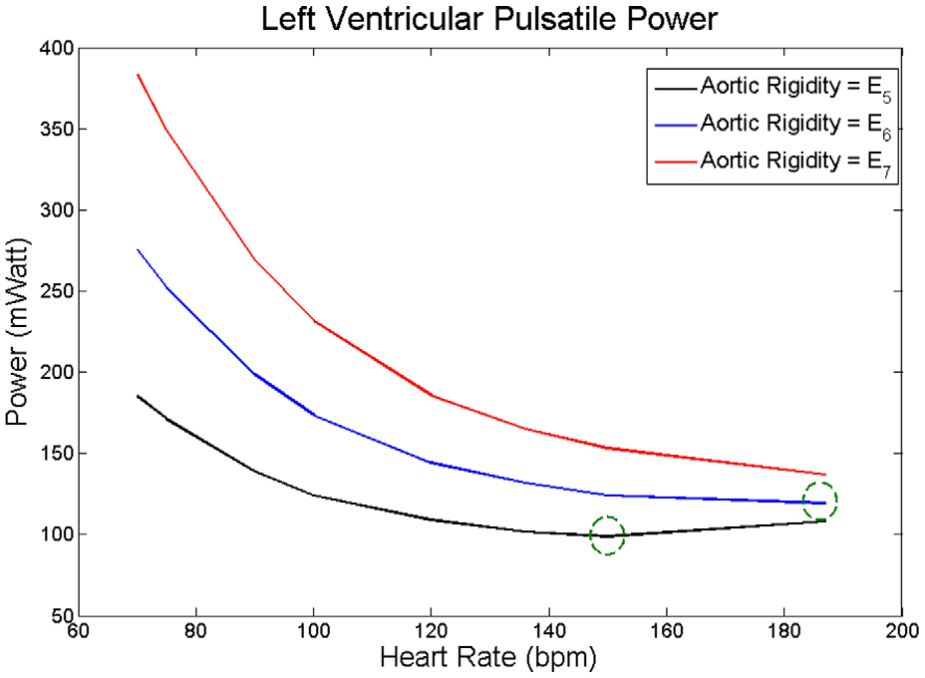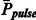 versus HR for extreme values of aortic rigidity. *E_5_ = 2E_1_* (black), *E_6_ = 2.5E_1_* (blue), and *E_7_ = 3E_1_* (red), where *E_1_* corresponds to the values given in Table 1. Green circles are the heart rates corresponding to the minimum 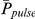 value at each level of aortic rigidity.

Figure 6 shows 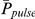 as a function of HR for three levels of aortic rigidity. As mentioned before, the cardiac output (average flow per cardiac cycle) is kept the same for all cases. As the HR increases, the 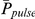 decreases until the HR reaches an optimum point where 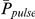 is minimized (and as a result 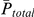 is minimized). The 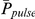 increases with HR beyond this optimum point. This phenomenon is present for all three cases. Interestingly, the optimum point shifted towards higher HR as aortic rigidity increased. In fact, as Figure 7 demonstrates, these phenomena still exist at very high rigidities, two- or three-fold greater than those of Figure 6.

## Discussion

The main findings of this study are as follows: (i) an increase in aortic rigidity leads to an increase in both the steady and pulsatile loads on LV; (ii) at higher aortic rigidities, the effect of pulsatile load becomes more dominant; (iii) at a given heart pumping condition (cardiac output and inlet flow wave), there is an optimum heart rate that minimizes the pulsatile LV power; and (iv) the optimum HR shifts to higher values as aortic rigidity increases.

Pulsatile load on the LV is the result of complex wave dynamics in the arterial network. As the largest and most compliant vessel extending from the heart, the aorta dominates the wave dynamics that the LV experiences. Although pulsatile load on the LV only accounts for a small portion of the total energy of the heart [27], [28], its adverse affect on the LV has been well accepted. Indeed, clinical studies have confirmed that abnormal pulsatile load plays an important role in the development of LVH and progression of LVH to CHF [4], [8], [9], [10]. Hence, aortic wave dynamics—as the determinant of LV pulsatile load—plays an important role in pathogenesis of LVH and CHF.

First, we investigated the effect of aortic wave dynamics on LV power requirements by changing aortic rigidity at a fixed HR( = 75 bpm) while keeping constant all the other aortic wave dynamic determinants such as CO, shape of inflow wave, peripheral resistance, terminal compliance, and the locations of the reflection sites. Both steady power and pulsatile power increase at higher rigidities. The increase in pulsatile load is due to both reduced compliance and wave dynamics.

Second, for a fixed cardiac output (CO = 4.6 *L/min*), we studied the effect of aortic wave dynamics on the pulsatile LV power requirement across a physiological range of heart rates. Our results reveal that there is an optimum HR (within physiological range) at witch pulsatile LV power requirement becomes a minimum, thereby confirming a prediction by O'Rourke [29].

**Figure 8:**
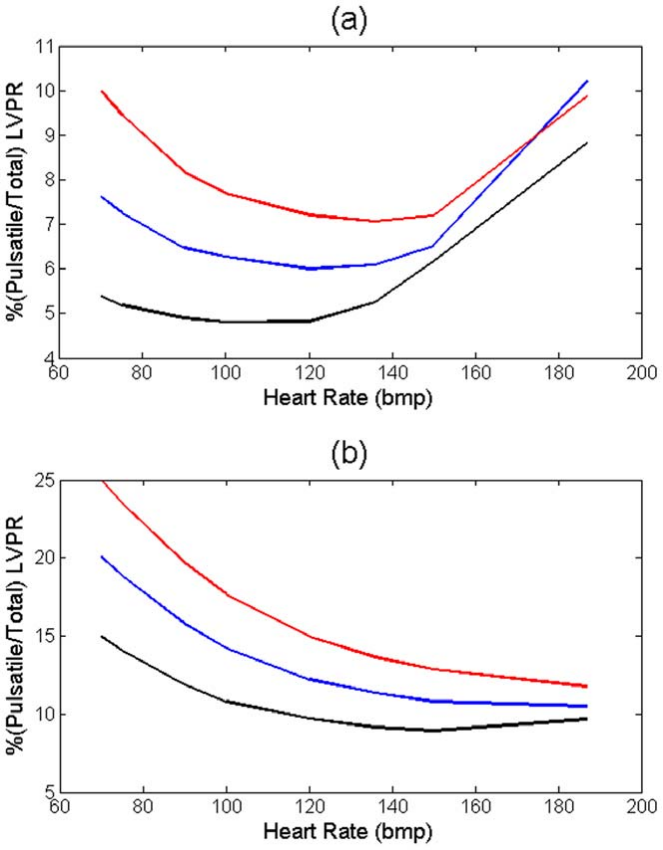
(a) Percentage of 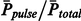 versus HR for the cases given in Figure 6; *E_1_* (black) corresponds to the values given in Table 1, *E_2_ = 1.25E_1_* (blue), and *E_3_ = 1.5E_1_* (red). (b) Percentage of 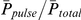 versus HR for the cases given in Figure 7; *E_5_ = 2E_1_* (black), *E_6_ = 2.5E_1_* (blue), and *E_7_ = 3E_1_* (red).

The pulsatile power continues to decrease with increasing HR until it reaches its minimum point. Beyond the minimum point, the aortic waves start acting destructively, and as a result, the pulsatile power starts elevating with the HR (see Figures 6 and 7). The same pattern has been shown in an animal study performed by O'Rourke [11]. He studied the affect of HR on the ratio of the pulsatile to total LV power. In Figure 1 of his paper, he showed the pulsatile/total percentage versus HR for 3 dogs (dog 7, 9, and 32) where dog 32 showed the same pattern as in Figure 5 and it had an optimum HR of around 120 bpm; however, he did not explain the existence of this minimum point in the paper. Notice that we sketch “pulsatile” load versus HR in figure 6 and 7. However, as Figure 8 shows, the shape of the graphs in Figures 6 and 7 will be preserved if one sketches the percentage of pulsatile load over total power versus HR.

### Clinical example: Smoking, Aortic Stiffness, and Heart rate

It has been shown in clinical studies that both aortic rigidity and HR are higher in habitual smokers compared to nonsmokers [10], [30], [31], [32]. It has also been shown that even smoking a single cigarette leads to a transient increase in pulse wave velocity (aortic rigidity), whether habitual or not [10], [33]. Hence in all cases the HR—even for habitual smokers whose HR level is generally higher—will increase during and immediately after smoking even one cigarette [34], [35]. We showed that the optimum wave condition leads to a minimum pulsatile load at a specific HR. Hence, our results suggest a possible explanation for HR elevation in smoking. As aortic rigidity increases—such as from short-term or long-term smoking—the optimum wave condition shifts to a higher HR and therefore the heart increases the HR to reach the new optimum.

Some may argue that increasing HR as a compensatory mechanism for decreasing LV pulsatile load can have a metabolic disadvantage for the heart in terms of increased myocardial oxygen consumption and impaired ventricular-arterial coupling. However, it has been shown in previous studies that under normal conditions and in the absence of heart diseases (e,g. dilated cardiomyopathy), increased HR may even results in enhanced LV systolic and diastolic performance [36], [37]. Furthermore, under normal conditions, the LV-arterial coupling remains optimal after a moderate increase in HR [38].

### Model limitation

In this study, we assumed that the heart acts as a flow source and hence specified the flow wave at the inlet. Although in general the heart is neither a flow nor a pressure source, the behavior of a normal heart is closer to a flow source [39]. In fact, in the case of a hypertrophied left ventricle (LVH condition), the heart acts completely as a flow source [39]. For a given metabolic condition, body requires a certain flow rate and therefore we found it reasonable to consider the heart as a flow source in our study.

Additionally, the aortic curve (arch) and the aortic branches were not included in our computational model (see figure 1). However, the influences of branches in terms of wave reflection were included in the outflow boundary condition of our model (by assuming that the branches produce summated reflected waves). Exclusion of the arch can be justified by considering the fact that the curve has an insignificant effect on pressure waves [15].

### Conclusion

Our results in this computational study show that at a given heart's pumping condition, there is an optimum condition for aortic waves that minimizes the pulsatile load (and consequently total workload) on the heart. In addition, based on clinical observations, our results suggest that the heart may use this fact as a temporary compensatory mechanism to reduce myocardial workload. Therefore, controlling and modifying aortic wave dynamics—as the determinant of LV pulsatile load—can be a therapeutic approach for the reversal of LVH and the prevention of HF.
